# Leveraging Social Computing for Personalized Crisis Communication using Social Media

**DOI:** 10.1371/currents.dis.b2c5870adf1b7a77af82e7d5552aabe7

**Published:** 2016-03-24

**Authors:** Dmitry Leykin, Limor Aharonson-Daniel, Mooli Lahad

**Affiliations:** Department of Emergency Medicine, Recanati School for Community Health Professions, Faculty of Health Sciences, Ben-Gurion University of the Negev, Israel; PREPARED Center for Emergency Response Research, Ben-Gurion University of the Negev, Israel; Department of Emergency Medicine, Recanati School for Community Health Professions, Faculty of Health Sciences, Ben-Gurion University of the Negev, Israel; PREPARED Center for Emergency Response Research, Ben-Gurion University of the Negev, Israel; The Community Stress Prevention Centre (CSPC), Israel; MA program in Dramatherapy, Tel-Hai College, Upper Galile, Israel

## Abstract

Introduction: The extensive use of social media in modern life redefines social interaction and communication. Communication plays an important role in mitigating, or exacerbating, the psychological and behavioral responses to critical incidents and disasters. As recent disasters demonstrated, people tend to converge to social media during and following emergencies. Authorities can then use this media and other computational methods to gain insights from the public, mainly to enhance situational awareness, but also to improve their communication with the public and public adherence to instructions.

Methods: The current review presents a conceptual framework for studying psychological aspects of crisis and risk communication using the social media through social computing.

Results: Advanced analytical tools can be integrated in the processes and objectives of crisis communication. The availability of the computational techniques can improve communication with the public by a process of Hyper-Targeted Crisis Communication.

Discussion: The review suggests that using advanced computational tools for target-audience profiling and linguistic matching in social media, can facilitate more sensitive and personalized emergency communication.

## ****Background****

The extensive use of social media in modern lives redefines social interaction and communication. Many of us are heavy consumers of the different channels of the social media, whether it is social networks, microblogs, photo sharing, forums, blogs or other types of the media. Nowadays, we are passive absorbers of information and active distributers of it. Many people spend a considerable portion of their time maintaining their virtual social networks, expressing themselves in different settings, staying tuned with the latest news and finding relevant information. During and following crisis or emergency situations, people turn to social media for various reasons, to make sense of the world through words, or as described by Lahad^1^ for the natural need of the storytelling animal'. We express ourselves and share our inner world through combination of words that others instantly absorb, process and act upon.

Disaster situations increase the need for information. The need, roots from high levels of uncertainty, true threats to life or health, and often manifest themselves in an urgent necessity for immediate information^2^. Effective crisis communications can therefore serve to mitigate anxiogenesis and direct rapid and focused rescue, recovery, and rehabilitative operations^3^. In the past, in such situations, the mass media disseminated messages and information to the public. Social media has revolutionized how individuals interact and how organizations and decision makers communicate with the public during routine and emergency times through the possibility of mass interactive communication. It has been suggested by practitioners that successful message phrasing, delivered to specific groups with specific characteristics, plays a crucial role in the communication process 4. While tuning to the public is a challenging task, the application of state of the art computational methods on the huge amount of user-generated textual content in social media, can enhance public understanding through insight extraction.

The current review seeks to clarify the following topics:

1. The role of social media in the process of crisis and emergency communication
2. The use of social computational approaches in the process of crisis and emergency communication.
3. The potential contribution of user-generated content and communication personalization to the effective management of exceptional or out-of-the-ordinary situations, otherwise known as emergency and crisis management^5^.

Finally, we will discuss where and how the introduction of new media and the advancement of social computational approaches calls for an update of theories and models of crisis communication.

## ****Literature Review****

**Social Media and Crisis**

The rise and proliferation of Web 2.0 applications revolutionized the way people interact and collaborate with each other. At the core of the Web 2.0 framework, are the social media, "a group of Internet-based applications that build on the ideological and technological foundations of Web 2.0, and that allow the creation and exchange of User Generated Content (UGC)"^6^. Social media nowadays includes collaborative projects, blogs and microblogs, content communities, social networking sites, virtual game worlds and virtual social worlds. Through these applications, users create online communities to share information, ideas, personal messages, and other content. This user-generated content in a changing and dynamic virtual community creates a massive and unstructured data^6^.

In modern society, the signiﬁcance of the social media in everyday life has increased dramatically, turning the world into a “global village”^7^. Since the late-20th century, new methods of mass media including the internet, cellular technologies and personal portable devices, have been considered as fulfilling a significant role in agenda setting and framing^8^, creating social capital^9^, and affecting politics, society and culture^10^. According to a recent report that summarizes social, digital & mobile statistics among global users^11^, 42% of world's population are active internet users, and above one-quarter have active social media accounts. With regard to social networking platforms, the report shows that there are 1.36 billion Facebook users, 629 Million Qzone users (Chinese social network), 343 million Google+ users, 300 million Linkedin users and 271 million Twitter users. Twitter, a microblogging communication platform, for instance, reaches an estimated total of over 50 million tweets per day^12^, and during crisis twitter activity increases exponentially^13^.

Social media has become an essential mean of communication during disasters and it has been suggested that best practices need to be established to assist organizations, government and decision makers in optimizing risk and crisis communication in this era 14. Houston et al.^15^ developed a framework for the practice and study of disaster social media. They note that disaster social media users in the framework include communities, government, individuals, organisations, and media outlets. Those players seek to gain insights from the massive user-generated content published in the communication channels, provided by the social media platforms. This published timely information is quickly spread across multiple social networks, and may affect, shape or direct public behavior without official involvement or even in contradiction to formal instructions 16. Although many challenges exist during social media message processing and analysis, (e.g. management of information overload, credibility issues, and prioritizing different classes of messages), state of the art computational methods exist to carry out complex information processing operations. 17

**Crisis Communication**

Crisis communication can be defined broadly as the collection, processing, and dissemination of information required addressing a crisis situation. Crisis communication is a research and practice field in various setting and crises; including national^18^, organizational crises^19^^,^ 20 , natural^21^ , man-made^22^ and technological disasters^23^. Effective communication with the public remains a crucial role in public safety through crisis preparation and response^24^. The media are critical for facilitating pre-disaster preparedness, disseminating warning messages of pending disasters, providing information to citizens about the disaster, and facilitating recovery efforts^25^. Moreover, according to Firestone & Everly^3^ , crisis communications can play an important role in mitigating, or exacerbating, the psychological and behavioral reactions to critical incidents and disasters. One important adjacent field of crisis communication is Risk Communication, defined as any purposeful exchange of information about health or environmental risks between interested parties (e.g. governments, agencies, media, citizens and more)^26^. Reynolds and Seeger^27^ prposed a merged and comprehensive approach called "crisis and emergency risk communication".

**Social Media as a crisis communication platform**

Social media has been radically changing the communication landscape over the past several years and as a result, crisis communication is undergoing substantial change^28^. It becomes clear that social media serves as ultimate space for crisis communication processes. Implementation of traditional crisis communication activities, was identified as one of the functions of disaster social media^15^. A recent survey^29^ of about 288 government officials in the US revealed that 71% of them were using social media during crisis, with Facebook as the popular social medium. Officials used social media during various types of crises, including public health^30^, natural disasters, transportational, political, social and criminal crises. Researchers found that the degree of social media use, rather than the number of tools used, was positively correlated with local city officials’ evaluations of their ability to control a crisis situation and the strength of their responses^29^.

Another research team^31^ systematically investigated crisis messages collected from Twitter. Relevant tweets for 26 different crisis situations that took place in 2012 were sampled from the available public twitter stream, and for each situation types of information and sources of that information were examined. During crisis situation, eyewitnesses, government, NGOs, business, media & news organizations and outsiders participate and publish different information types in social media 31. Types of information include data about affected individuals (e.g. people trapped, casualties, people missing, found or seen), infrastructure & utilities (e.g. damages, reports about environment, and availability of services), donations & volunteer (e.g. donations of money, goods of services; requesting help; shelter needed, food shortage/distribution; volunteer information); caution & advice (e.g. warnings, preparation; caution & advice; tips; safety), sympathy & emotional support (e.g. concerns and condolences; gratitude, prayers; support; emotion-related info) and other useful information (e.g. flood level; weather, wind, visibility; information verification). Based on the available data of that study (http://crisislex.org/tweet-collections.html), it seems that the Government, being the official source of information during crisis, was responsible for less than 5% of all published information. Of the applicable information types, the majority (32%) of the communication efforts by the government were about useful information (32%) and caution and advice (27%). Only 6% of the published tweets concerned about sympathy and support.

**Effective Crisis Communication.**

Seeger^32^ demonstrated ten best practices of crisis communication, drawn from a literature review and verified by expert crisis communication panel that reached high consensus. Seeger^32^ divides the recommendations into three broad categories: strategic planning, proactive strategies and strategic response. These are used as principles or processes that underlie an effective crisis communication plan and an effective crisis response.

Covello, McCallum, & Pavlova^33^ note that detailed, in-depth knowledge and understanding of both the characteristics of target audience and the community in which the target audience resides, are needed for effective message development. Target audience characteristics include knowledge, attitudes, perception, behavior, beliefs, values, needs and concerns, while community characteristics include information about social networks, opinion leaders and community dynamics. Covello^34^ suggested several risk communication templates to utilize in the process of risk communication: 1) CCO (Compassion, Conviction, Optimism) template, which is particularly useful when responding to a question indicating a high level of emotion or outrage^4^. 2) Primacy/Recency template^35^ emphasizes the first and last messages in the communication due to the restriction of recall of information. 3) 27/9/3 template states that the combination of the three key messages should equal a total of 27 words, 9 seconds spoken aloud, and 3 key messages^34^. 4) AGL-4 (Average Grade Level minus 4)^4^ template recommends phrasing the message at four reading grade levels below that of the stakeholder – taking into account the national reading grade level. 5) 1N=3P (Negative equals 3 positives) template counterattacks the weight of negative messages by introducing three positive messages for every negative message. 5) TBC (Trust, Benefits, Control) template suggests to phrase three messages that are phrased with specific order and content (trust first, benefits second and control last). These templates, however, were not empirically tested and thus limit our certainty regarding effective messaging.

The media plays a crucial role in shaping public response to terrorism and other disasters^25^. Effective crisis communication suggests the need of an unhindered but purposeful exchange of information within and between authorities, organizations, media, involved individuals, and groups before, during, and after a crisis^36^. Covello et al.^33^ also argue that risk communication is no longer a neglected topic within government. With the changing media environment and the developing online atmosphere, traditional media are shifting to practices that are more adapt to a social media environment^37^. Furthermore, to improve services and communication with the population, government officials seek to leverage these new media channels. Nevertheless, Graham, Avery & Park^29^ emphasize that government’s engagement through social media should be more active and reflect a clear response priority in crisis communication plans. Ambiguous or unreliable communications can cause damage and serve to exacerbate publics’ mental health reactions and a delay in operational response and recovery^38^ ****To conclude, it is important to incorporate the use of citizen generated content into any crisis plan and learn to respond to the media and public even quicker than before^39^

**Models and theories of Crisis Communication. **Over the years several models of crisis communication were developed^40^, such as the Situational Crisis Communication Theory (SCCT^41^). SCCT "predicts the reputational threat presented by a crisis prescribes crisis response strategies designed to protect reputational assets"^41^, especially in organizational context.

Chaos Theory^42^ was described as a general framework for understanding crisis communication^43^. CT argues that chaos or disorder may be the necessary precursor of a higher level of order. CT functions best at the broad level of a paradigm for understanding the behavior of complex systems. Seeger^43^ adds that in the context of CT, small variance in communication processes, message phrasing, distribution, timing or other factors may produce extensive fluctuations in systems, leading to bifurcation.

Reynolds & Seeger^27^ presented the Crisis and Emergency Risk Communication (CERC) five-stage integrative model. The model blends crisis and risk communication together into a processes model (see^27^), assuming that crises will develop and progress in a predictable and systematic ways. According to the working model of CERC, communication processes occur during five stages: 1) Pre-crisis, 2) Initial Event, 3) Maintenance, 4) Resolution and 5) Evaluation. In each stage, communication can have different aims, strategies and target audiences.

With the advancing new media, the social media aspect was integrated in these models. For instance, the Social Media Audience Sharing Model (SMA)²^24^ aims to increase the reach for messages disseminated via a given social media platform in the context of an emergency. Social mediated crisis communication model (SCCM)^44^ is another model that serves as a framework for crisis communication management in the changing media landscape and explains how the source and form of crisis information affect organizations response options and provides recommended social-mediated crisis response strategies. The Networked crisis communication model^45^ examines the influence of communication strategy and media type on damage to reputation, as well as secondary crisis communication and secondary crisis reactions. Although these models recognize new players in the crisis communication processes (e.g. influential social media creators, follower, and the growing empirical evidence that emphasizes the psychological functions of social media during emergency management - during most of the time, less or no consideration is given to psychosocial aspects during crisis communication. The need to focus on other participants (such as the public) rather than the organization or the responding authority, is echoed in Liu & Fraustino^46^ who suggests that scholars should move beyond predominantly focusing on image management, emphasized by dominant crisis communication theories

**Accommodated Communication**

Considerable research, using text derived from social interactions, such as natural conversations and social media conversations, suggests that individuals tend to converge in various dimensions such as posture, pause length, utterance length, self-disclosure, head nodding, backchannels and linguistic style^47^. Niederhoffer & Pennebaker^48^ studied psychometric properties of language in dyadic interactions and assessed the degree to which people coordinate their word use in natural conversations, derived from internet chat and laboratory conversations. Based on a text-analysis software, they found that individuals in dyadic interactions exhibited linguistic style matching (LSM) on both the conversation level as well as on a turn-by-turn level. LSM found in research to be a predictor of social dynamics in small online and face-to-face groups^49^. LSM, also found to be a robust marker for romantic relationship stability, stressing the importance of similarity in the way people converse with each other in the context of interpersonal processes^50^. Lord, Sheng, Imel, Baer & Atkins^51^ saw that’s language style synchrony between client and therapist was predictive of empathy ratings during evidence-based behavioral treatments like motivational interviewing (MI). Therefore, it is apparent that synchronized verbal behavior holds an important role in various interpersonal interactions.

LSM, in fact is a derivative of Communication (or speech) Accommodation Theory (CAT), an intergroup theory of interpersonal communication, provides a framework for understanding how and why people adapt their communication toward and away from others and the social consequences of doing so. The theory holds that people tend to preform accommodative and non-accommodative moves, to reduce or increase significant social distances between the speakers, depending on the different circumstances. Convergence and divergence, called in the CAT approximation strategies, include the adaptation (or the alteration) of communicative behaviors in terms of wide range of linguistic-prosodic-nonverbal features. The theory also proposes different patterns of accommodation (upward vs. downward, and symmetrical or asymmetrical), based on the reciprocity of the speakers^52^. During the last years, examination of CAT progressed to the domain of electronic communication, such as e-mail, text messages, voice mail and recently also was adapted to electronic communications and the social media sphere. Danescu-Niculescu-Mizil et al.^47^ examined and verified the hypothesis of accommodated communication in the context of twitter conversations. The researchers developed a probabilistic framework that enabled to model accommodation and measure its effects. They focused on linguistic style feature, derived from the Linguistic Inquiry and Word Count^53^.

To the best of our knowledge, CAT was never integrated in any crisis communication models published in the academic literature. The closest domain LSM, was mainly researched in the context of crisis negotiations of suicidal and surrender outcomes, and hostage taking negotiation outcomes^54^^,^ 55 ^,^ 56. In these studies, the researchers analyzed the correlations between the linguistic behavior of each communicator across 18 linguistic dimensions, like word count, prepositions, negations, emotionally toned words and others. LSM was assessed by the strength of the correlation and averaged across all conversations taken into account. The studies showed that negotiators (police officers and hostage takers) tended to show greater levels of linguistic style matching in successful negotiations compared to unsuccessful negotiations^57^, and an overall consistency in the linguistic behavior of subjects and police negotiators in surrender and suicide incidents^54^.

Interestingly, linguistic matching or accommodated communication is not emphasized in the processes models of crisis and risk communication, and possibly is taken for granted. Even when a certain aspect that is related to crisis communication with the public is presented, such as crisis communication strategies from the Situational Crisis Communication Theory^41^, linguistic features and linguistic accommodation are not explicitly mentioned as being part of the processes. Some research considered other aspects like number of words, number of sentences and words per sentence^58^. In other words, when a crisis communication model, like the networked crisis communication model^45^ and social-mediated crisis communication model^59^, mentions elements like message form or message strategy, it ignores the linguistic characteristics of the message. The best practices in crisis communication^32^ introduce general principles of crisis communication, such as strategic planning, proactive strategies & strategic response, and each include sub categories that can be directly related to public communication, e.g “be open and honest”, “communicate compassion” and “provide self-efficacy”. Since speakers’ verbal style also influences how messages are perceived^60^, we suggest that even if crisis managers will act according to these principles, they should employ certain linguistic styles and consider delivering messages in public accommodated language

**Social Computing**

"Computational social science is an emerging research area at the intersection of computer science, statistics, and the social sciences, in which novel computational methods are used to answer questions about society"^61^ (p. 257). Ericson^62^ argued that social computing refers to "systems that support the gathering, processing and dissemination of information that is distributed across social collectives. Furthermore, the information in question is not independent of people, but rather is significant precisely because it linked to people, who are in turn associated with other people." Scholars^63^ stated that social computing represents a new research frontier for information systems. Social computing environments present settings for data collection on a wide variety of aspects for researchers interested in online behavior of individuals, both in natural observations and for controlled experiments.

**Behavioral Targeting.**

Behavioral targeting (BT), also called online profiling^64^ or hypertargeting^65^ uses historical user behavior to predict user behavior and affinities in web applications such as targeting of online advertising, content personalization and social recommendations^66^. BT is used by online advertisers to increase the effectiveness of their campaigns, and is playing an increasingly important role in the online advertising market^67^. Through series of experiments, compared to standard run of network advertising, BT advertising was found to be more successful, creating greater utility for consumers from more relevant advertisements and clear appeal for advertisers from increased ad conversion^68^. User profiling is performed using novel computation techniques^66^^,^ 69, commonly fall inside the social computing paradigm, an approach to analyze and model social behaviors on different media and platforms^70^. Van Dam & van de Velden^71^ proposed that social networks, like Facebook, can be “operationalized to gain insight into the individuals connected to a company's Facebook site” (p. 60). In their study they describe a user profile data collection framework that uses “Facebook insights” (accessed by the admin) and other personal public information on Facebook to cluster users. They propose that their methodology can be implemented into an analytical customer relationship management (CRM) framework aimed at the analysis of customer characteristics that may help improve a firm's customer management strategies.

**Recommender systems**.

Ricci, Rokach, & Shapira^72^ defined Recommender Systems (RS) as “software tools and techniques providing suggestions for items to be of use to a user” (p. 1). They add that since recommendations in most cases are personalized, different users or user groups receive different suggestions. RSs try to predict what the most suitable products or services are, based on the user’s preferences and constrains (learned and collected from users. Many product purchasing sites like Amazon and E-Bay use advanced recommendation engines^73^, but social networking sites (e.g. Facebook) use RS as well, to “push” relevant social content based on use preferences, actions and texts^74^.

Recommender systems in Emergency**. **In the context of emergency, the existing published literature suggests that recommendation systems can be integrated for improved disaster management^75^, Construction and real estate crisis management^76^ and supply distribution in emergency^77^. However, we could not find existing literature of how crisis communication can be aided by RS. More specifically, how crisis messages are selected for individuals or groups of individuals based on some characteristics. Research shows that integrating data obtained from popular social media networking websites significantly can improve results of existing recommendation systems^78^.

**Computational social science in the face of disasters.**

Over the past years technologies that rely on citizen sensing have been playing a major role in real life applications, such as public and environmental health surveillance^79^, and other participatory social activities^80^^,^ 81 ^,^ 82. Purohit, Castillo, Meier & Sheth^83^ noted that with the explosion in social media and the universal mobile access– researchers have unique opportunities to "extract social signals, create spatial-temporal mappings, perform analytics on social data, and support applications that vary from situational awareness during crisis response, preparedness and rebuilding phases to advanced analytics on social data, and gaining valuable insights to support improved decision making". (p. 1).

**Social computing, analytics, visualization for crisis communication.**

In the era of big data analytics and social media, incorporation of business intelligence systems in the organization are very common. These systems are responsible for data processing, analysis and visualization for better decision making and business function. Based on a recently published taxonomy in the domain of text analytics and visualization^84^, one can find various analytical tasks and visualization tasks with specific aim. The tasks are common text analytical procedures^85^^,^ 86, and include: 1) Text Summarization / Topic Analysis / Entity Extraction, 2) Discourse Analysis; 3) Sentiment (Opinion) Analysis, 4) Event Analysis; 5) Trend / Pattern Analysis; 6) Lexical / Syntactical Analysis; 7) Relation / Connection (Association) and 8) Translation / Text Alignment Analysis. These tasks later serve for visualization procedures for: 1) Region of Interest, 2) Clustering / Classification / Categorization, 3) Comparison, 4) Overview; 5) Monitoring; 6) Navigation / Exploration; 7) Uncertainty Tackling.

Tables 1 & 2 summarize current state of the art of how social computing and text analytical tools can be integrated in the best practices of crisis communication^27^^,^ 32. We focus on the objectives in the processes of crisis and emergency communication^27^ that occur in different times of the crisis life cycle and have direct association to communication processes with the public. Finally, we describe how computational and analytical tools can be utilized to accomplish these aims.

**Figure:**
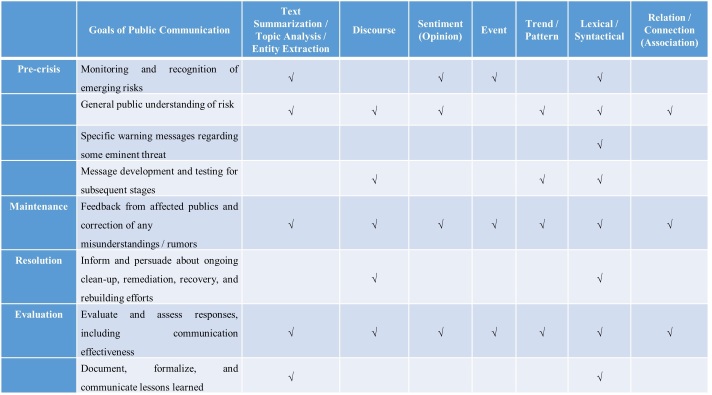
**Table 1: Analytical Tasks in the domain of Text Mining & Analytics for Crisis and Emergency Communication using Social Media**

**Figure:**
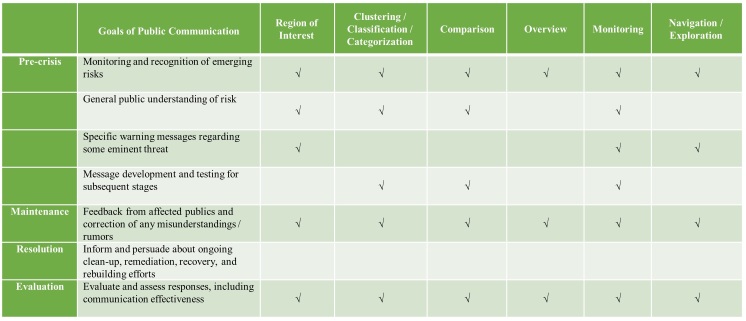
**Table 2: Visualization Tasks in the domain of Text Mining & Analytics for Crisis and Emergency Communication**

**Monitoring and recognition of emerging risk**.

Information extraction tools using pre-defined lexicons of crisis related terms^87^^,^ 88, as well trend analysis (e.g. hashtag or term anomaly detection) could be a useful way to spot potential risks in the environment, based on social media feeds and streams, that previously have shown themselves to be valuable sources of real-time information about what is happening in the world^89^. Social Media feeds can represent a hybrid form of a sensor system that allows for the identification and localization of the impact area of the event^90^^,^ 91. Next, using text visualization techniques, geo-located information can be mapped and depicted in a way a potential risk can be categorized according to its geographical occurrence. As people will explicitly mention different terms related to potential risks, dynamic map would show categorizations of the terms into risk topics, allowing crisis managers to monitor risks.

**General public’s understanding of risk.**

Public’s knowledge of risk is conducted most of the times through surveys^33^. However, it can also be inferred from social media discussions. Emergency authorities can, for instance, publish question regarding different issues related to specific risk in their feed (e.g. lifesaving behavior in wild-fire) and later investigate the discussion followed by post publishing. Research shows that social media discussions tend to be topic dependent^92^^,^ 93, especially when the page posts direct questions and asks the general public to discuss an issue. Crisis managers could train text responses over documents that an expert panel would consider as relevant to the original question (e.g. “what is the correct lifesaving behavior during wildfire”), and also classify documents according their answer correctness using Natural Language Processing (NLP)^94^ and classification algorithms^86^. Overview level of the public understanding of risk, based on social media discussion, then could be visualized using simple pie charts.

**Message development in crisis communication.**

Based on the variety of approaches presented above, this aspect can be aided by discourse analysis^95^, linguistic analysis and natural language processing. Using lexical approach^96^ and psycholinguistic tools^53^, crisis managers can analyze messages from different perspectives– from syntactic features of the text and various word meaning categories (e.g. LIWC categories). We propose that integration of such tools would enable to observe whether all pre-designed crisis messages are adapted to the target audience’ communication capacities.

**Affected public opining mining.**

To gain feedback from the affected public, one should monitor social media and extract signs from citizen who speak and discuss the emergency. Temnikova et al.^88^ presented a terminological resource, EMTerms, which include over 7,000 terms used in Twitter to describe various crises, classified into 23 information-specific categories (e.g. caution and advice, infrastructure damage, supplies needed or offered, personal updates, safety and security and more). Such resource can assist decision makers monitor different categories of information, and later communicate to the public on the most prominent information. Once information is retrieved, automated text analytical approaches, like opinion mining, can assist in capturing public's perceptions. Opinion mining refers to the extraction of emotion, appraisal and opinion words that are associated with certain social issue, people or entity (e.g. product, public figure, and event) and the classification of the words into different opinion categories (e.g. positive vs. negative, support vs. against)^97^. Seeger^32^ argued that capturing public's perceptions are important due to the tight association between beliefs and actions. He adds that monitoring public's risk perceptions and opinion prior and during crisis is essential for crisis response and message adaptation to public's needs and concerns. This text analysis procedure has various application in a review summarization & classification, market and brand analysis^98^, political opinion analysis and decision making. Opinion and sentiment classification is performed on a document and sentence levels by extracting relevant features (e.g. single and/or multiple words, emoticons) from the text and applying computational techniques for estimating the overall polarity or direction of the text. The most common methodology involves pre-defined sentiment or opinion lexicons that carry thousands of domain-specific words, or machine learning techniques that use supervised learning for sentiment classification.. In the context of natural/human made disasters, the assessment of sentiment is somehow scarce. Nagy, Valley, & Stamberger^99^ compared methods for evaluating sentiment in disaster microblogs and explored patterns of change in emotion of the crowd during a technological disaster. Unfortunately, assessment of sentiment towards the general crisis event provides only partial information for crisis communication practitioners regarding crowd opinion, since it is not matched to specific risk or issue, and more

In the case of health crisis and pandemics, however, considerable research is found. For instance, in the case of swine flu pandemic, Salathé & Khandelwal^100^ used publicly available data from users of online social media and measured spatial-temporal sentiment towards a new vaccine over a period of six months. They further found strong correlation between sentiments expressed online and CDC-estimated vaccination rates by geographical region. This provides good example of how policy makers can use publicly available data to learn about public's perception regarding risk and its' related behaviors.

**Misunderstanding/Rumors Corrections**.

Possibly one of the most researched topics in the domain of disaster computing and social media research during crisis, is information credibility^101^^,^ 102. Castillo et al.^102^ analyzed information credibility of news propagated through Twitter and suggested that by using automated methods a tweet can be classified with up to 80% precision as being credible or not. Their automatic credibility estimation was done by extracting message-based features (e.g. text length, sentiment ratio in the text, inclusion of hashtag), user-based features, topic-based (e.g. aggregates computed based on message and user based features), and propagation-based features (e.g. depth of a retweet in the network of message spread, initial tweets per topic).

**Evaluat** **ion and assessment of responses, including the effectiveness of communication.**

Similarly to the previous section, evaluation of public responses can be achieved through continuous social media monitoring using opinion mining, to infer on concerns, panics, and the emotional impacts of interactions among social media users^103^. Recent studies demonstrated capabilities to track changes in sentiments of affected public during natural disasters^99^^,^ 103 ^,^ 104.

## Proposed Process of Hyper-targeted Crisis Communication in Social Media (HCCS)

In the above paragraphs, we described how advanced analytical tools can be integrated in the processes and aims of crisis communication. We suggest that the availability of the computational techniques can improve communication with the public by a process of hyper-targeted crisis communication. Figure 1 demonstrates how the data posted and shared through social media is collected for monitoring, analyzed and prepared for a tailor-made crisis communication.

**The Process of Hyper-targeted Crisis Communication in Social Media (HCCS):**
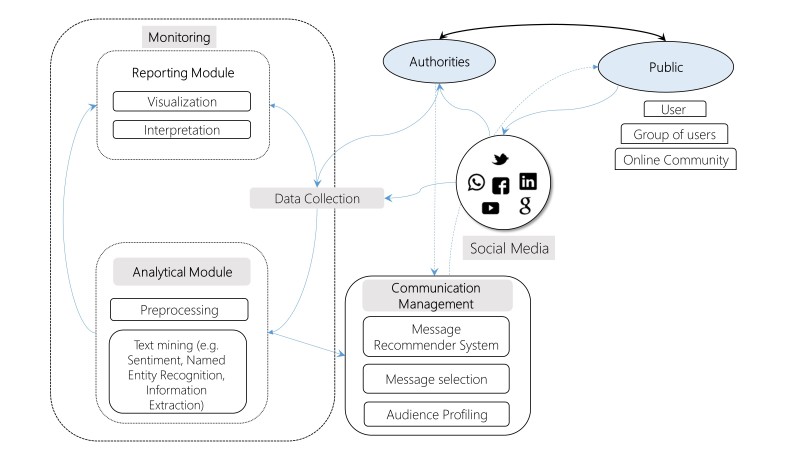

In the process, massive amounts of textual user-generated content in social media is continually generated and monitored for crisis or emergency related communication. Once detected, content is collected and transferred to the analytical phase, where it is preprocessed, analyzed according to various text mining analytical methods. The information then is either transferred for reporting where it can be visualized and interpreted or moves to the communication management module. Authorities, crisis managers and designated spokespersons personnel can communicate with the public, using the communication management module, which include a recommender system that proposes matched messages. This sub-system is also responsible for target audience profiling (i.e. hypertargeting), that enables accommodated communication to specific groups in the population. Bi-directional communication is then monitored for matching accuracy and secondary messages in the communication. During the conversation, a crisis messaging recommender system formulates messages based on conversation history and the other available parameters.

**Benefits of Targeted Crisis Communication**

Automatic categorization of unstructured vital information is of high importance for speeding up disaster management. More precisely, psychosocial information extraction is valuable for decision makers to understand the endurance of the public, public’ psychological needs and psychological risk, using validated theoretical models in the field of disaster psychology^105^. Being able to capture these aspects, emergency authorities can be more synchronized with population needs and thus more effective in their response to the public during disaster. By integrating disaster related psychosocial aspects in a command and control disaster management systems, they will be in a better position to manage the situation and speed up recovery.

Successful detection of language choice patterns from social media content may assist in guiding the authorities and service providers to respond on the same communication “channels” of the public, and enhance responsiveness and interactivity. This can further lead to a more cooperative public and enhanced public morale. It is our assumption that such focused messaging may not only improve the reception of the message, but also contribute to the ensuing behavior or compliance which is critical in disasters.

**Limitations of the present review and currents challenges**

One should remember that beyond text, additional forms of data are published on social media, sometimes far more influencing, since pictures (regular photo or meme) may generate more engagement than other forms of data (including text and video)^106^. Research documents applicable tools for multimodal analysis of social media content, that include both textual and visual data, e.g. Flicker photos, YouTube videos and more^107^. Thus, more precise public monitoring could be accomplished when integrating data from different modalities and content qualities, as adjacent task in the crisis communication processes. The above literature focused mainly on text that appears in the social media, that can be extracted and analyzed. As for now, some social media platforms offer easy means of extracting data, like the Twitter Streaming API^108^. One limitation in social media analysis is the inaccessibility to Facebook public feed API, which is restricted to a limited set of media publishers. This limitation is restricting research institutes and companies to obtain public posts, in a similar way that is available in Twitter. It is possible, though, to extract data from Fan Pages and Groups using publically available applications, like NetVizz^109^.

Decision makers should not solely rely on text analytics in social media when doing crisis communication. Though social media is very popular and has very high penetration rate^11^, some social and age groups in the population are not connected to the internet^110^. As this population tends to receive the information and guidelines during real time emergency via TV, radio, newspaper and word-of-mouth -the instructions will still need to use traditional means of mass communication..

Despite recent anecdotal reports indicating that authorities (e.g. Home-front command) increase their presence in the social media and continuously interact with the public in times of calm and emergency, still authorities prefer to communicate with the public in non-interactive means. This was previously mentioned as an obstacle for crisis communication^111^ in light of the raising of social media .Nonetheless, decision makers should not solely rely on methods of text analytics and data mining in social media when doing crisis communication and follow a well-organized risk and crisis communication plan^34^.

It is almost impossible not to consider privacy issues when discussing targeted communication^112^. Collecting information about the behavior of social media users, for research or commercial purposes, is considered invasive by the public as well as inappropriate^113^. Recently, the use of Facebook as a research platform for massive social experiment^114^, was criticized for not passing the ethical board and not providing informed consent to the participants. Conversely, Facebook advertising system is using profile information and interests (extracted from activity patterns of the users), to deliver targeted messages. Hence, authorities have to deal with this issue using sound legal advice and should address the privacy issues in their communications with the public to build trust and credibility.

## Recommendations

Both user profiling (i.e. behavioral targeting) and recommender systems are closely related. In both processes, user preferences are used for content recommendation. However, the application of behavioral-targeted recommendation system in the field of crisis communication is novel and deserves future studies. We propose that behavioral-targeted recommendation systems in the domain of crisis communication would be applied on two levels; public level and decision maker level. On the public level, personalized and adapted crisis messages would pop-up or appear in popular websites and social media based on two possible parameters: 1) the written content people provide on these sites (e.g. users’ public posts, reviews, and participation in talkbacks; 2) user behavior on site – such as “like”/”share’ or “re-tweet, or other forms of ratings. This approach would be effective if the user is active and engaged, but also may be relevant for passive users that mainly consume content rather than commenting on it. This proposal will be feasible in social media platforms which allow the tracking of users or has an agreement for data sharing with authoritative bodies. On the decision maker level, designated spokespersons would be assisted by the messaging recommender system to adapt their linguistic style to the user who approaches them (or vice versa) thus "tailoring" the response and assisting people in real-time to get the appropriate help, directions or support.

While certain online platforms for communication with the public in emergency were proposed^111^^,^ 115 they all significantly lack the user-profiling element, that might be in practice, an important factor in communication efforts^116^. Future web-based or mobile application-based platforms for disaster risk communication should include user-profiling module, based on different parameters extracted from users’ published text and data. We suggest social media management tools^29^ as suitable platform for crisis communication, as some of the tools enable the integration of extensions and apps that assist in social media data extraction, analytics and interpretation.

## Conclusions

In very fast pace, social media have acquired a prominent role in media and our daily life^71^. During crisis and emergency, people tend to approach social media not only because of the need for quick information, but also due to the human tendency for storytelling, which allows people to experience their lives as coherent, orderly and meaningful. It is what makes people’s life more than a blooming, buzzing confusion^117^. Communication with public during emergency is critical for successful emergency management^118^ life saving, rescue and recovery. On the other hand , one should take into account that 2.08 billion people world-wide are social media users^11^ and significant number of people are expected to participate in the online convergence, posting massive amount of user-generated content making it an almost impossible web of information. Using state of the art computational tools makes this mission possible. Being one of the fundamental tools of emergency management^118^, crisis and risk communication play an important role in mitigating, or exacerbating, the psychological and behavioral reactions to critical incidents and disasters. Thus the task of continues monitoring of the social web for crisis related information can significantly improve the efforts of emergency management. This is the reason for officials to be eager to exploit social media content to gain insight from the public for the optimization of risk and crisis communication^119^. Risk and crisis communication’ best practices are well documented and include various tasks during different crisis stages (e.g. understanding public’s characteristics, monitoring emerging risk, feedback from affected publics etc.). These aims and practices can be improved and become efficient when utilizing linguistic computational tools that can quickly assist decision makers in designing emergency campaigns, or/and managing communication with the public during emergency. Officials should also remember that the most effective communication are those designed for a specific audience^33^. Research shows that interpersonal communication that is linguistically matched between the communicators is not only more successful, but also reflects our natural tendency to adjust ourselves to the listener. Finally, decision makers and crisis managers need to acknowledge the potential of the massive generated textual information published during times of emergency in the social media. This knowledge is not only beneficial for situational awareness but also for user profiling, which in our case, is not intended for marketing purposes. Behavioral analyzed information from text posted on social media can leverage targeted communication during crisis, by automatic target audience characterization and adapted, psycholinguisticly matched risk message preparation. Thus, as we keep facing major disasters and emergencies that have became more frequent during the last decade, governments can be more effective in transmission and dissemination of warning messages or/and survival information, better manage different public reactions triggered by the crisis, gain credibility from the public, and improve public cooperation during emergencies.

## Competing Interests Statement

The authors have declared that no competing interests exist.
